# Intra-arterial transplantation of autologous mesoangioblasts in m.3243A>G mutation carriers is safe: First phase 1/2 human clinical study

**DOI:** 10.1016/j.ymthe.2025.07.005

**Published:** 2025-07-17

**Authors:** Florence H.J. van Tienen, Janneke G.J. Hoeijmakers, Christiaan van der Leij, Erika Timmer, Nikki Wanders, Patrick J. Lindsey, Fangzheng Yi, Fong Lin, Susanne P.M. Kortekaas, Helene Roelofs, Inge M. Westra, Pauline Meij, Lambert A.C.M. Wijnen, Irenaeus F.M. de Coo, Hubert J.M. Smeets

**Affiliations:** 1 Department of Translational Genomics, Maastricht University Medical Centre+, Universiteitssingel 40, 6229 ER Maastricht, the Netherlands; 2 Mental Health and Neurosciences Research Institute (MHeNS), Maastricht University Medical Centre+, Universiteitssingel 40, 6229 ER Maastricht, the Netherlands; 3 Department of Neurology, Maastricht University Medical Centre+, P. Debyelaan 25, 6229 HX Maastricht, the Netherlands; 4 Department of Radiology, Maastricht University Medical Centre+, P. Debyelaan 25, 6229 HX Maastricht, the Netherlands; 5 Research Institute for Oncology and Reproduction (GROW), Maastricht University Medical Centre+, Universiteitssingel 40, 6229 ER Maastricht, the Netherlands; 6 Center for Cell and Gene Therapy (CCG), Leiden University Medical Center, Albinusdreef 2, 2333 ZA Leiden, the Netherlands; 7 Department of Rehabilitation Medicine, Maastricht University Medical Centre+, P. Debyelaan 25, 6229 HX Maastricht, the Netherlands; 8 Milocron Therapeutics B.V., Oxfordlaan 70, 6229 EV Maastricht, the Netherlands

**Keywords:** phase 1/2 clinical trial, autologous cell therapy, m.3243A>G mutation, mesoangioblasts, mitochondrial myopathy, myogenesis, advanced therapy medicinal product, ATMP

## Abstract

Progressive myopathy and exercise intolerance significantly impair quality of life in over 50% of m.3243A>G mutation carriers, with no curative therapy currently available. We hypothesize that intra-arterial administration of autologous, mtDNA mutation-free myogenic stem cells, mesoangioblasts, can reduce mutation load, enhance oxidative phosphorylation, and improve muscle function. To test this, the tibialis anterior muscles of three m.3243A>G mutation carriers were damaged by eccentric exercise before the infusion of 50 million/kg autologous mesoangioblasts into the left anterior tibial artery. The right tibialis anterior muscle served as control. Expanded mesoangioblasts had a mutation load of <15%, although culturing increased this by 7%–15%. Infusion caused mild, transient discomfort without serious adverse events or vascular obstructions, as confirmed by angiography. Blood and muscle biopsies revealed no systemic or local inflammation at 24 h and 4 weeks post-transplantation. Biopsies of the treated muscle suggested mesoangioblast migration and early signs of regeneration. This first-in-human study demonstrates that intra-arterial administration of autologous mesoangioblasts is safe, with promising, although inconclusive, evidence for muscle regeneration and mesoangioblast homing. These findings support further investigation into the therapeutic potential of mesoangioblasts for treating myopathy in m.3243A>G mutation carriers.

## Graphical abstract

**Figure:**
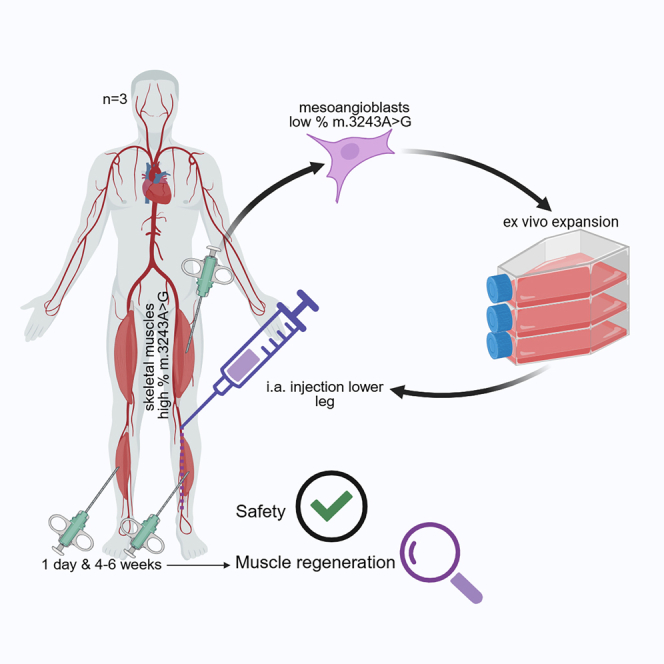

## Teaser

This first-in-human study demonstrates that intra-arterial administration of autologous mesoangioblasts is safe, with promising, although inconclusive, evidence for muscle regeneration. These findings support further exploration of stem cell-based therapies to reduce mitochondrial mutation load and improve muscle function in mitochondrial myopathy, addressing a major unmet need in this patient population.

## Introduction

Mitochondrial myopathies (MMs) represent a spectrum of progressive muscle diseases caused by compromised energy production in the mitochondrial oxidative phosphorylation system.^1^ In 20%–25% of the cases, MMs are caused by a heteroplasmic mutation in mitochondrial DNA (mtDNA),^2^ which is characterized by the co-existence of both wild-type and mutated mtDNA copies. One of the most frequent heteroplasmic mtDNA mutations is the m.3243A>G mutation in the dihydrouridine loop of the mitochondrially encoded tRNA Leucine 1 (UUA/G) gene.^3^ Skeletal muscles are commonly affected in symptomatic m.3243A>G mutation carriers. More than 90% of m.3243A>G patients display ragged-red fibers, and lactic acidosis in muscle and progressive myopathy and exercise intolerance occur in more than half of the m.3243A>G patients with a mean heteroplasmy level of ∼50% (range 30%–74%) in skeletal muscle.^4^^,^^5^^,^^6^ Age-related muscle loss even worsens the clinical manifestations, since the percentage of individuals with myopathy and severity of myopathy among m.3243A>G patients drastically increases with age and has the highest incidence in the fifth decade of life.^5^ Severe fatigue complaints were also reported by the majority (77%) of clinically affected m.3243A>G mutation carriers.^7^ Patients report muscle fatigue and weakness as having the greatest impact on quality of life.^8^^,^^9^ Consequently, treating the muscle-related complaints, irrespective of other possible disease manifestations, would be highly beneficial for patients.

Myogenic stem cell-based therapies are highly promising to combat myopathy and exercise intolerance. Myogenic stem cells called mesoangioblasts (MABs), are currently the only myogenic precursors that fulfill all criteria to be used as an advanced therapy medicinal product (ATMP) for systemic treatment, namely good *ex vivo* proliferation capacity, high myogenic capacity, and the ability to cross blood vessels, allowing intra-arterial (systemic) delivery to the affected muscle. Successful preclinical studies showed that treatment with three doses of 50 million/kg *ex vivo*-expanded MABs resulted in significant numbers of dystrophin-positive muscle fibers and clinical improvement in mice and dog models of Duchenne muscular dystrophy (DMD).^10^^,^^11^^,^^12^ Subsequently, intra-arterial delivery of allogeneic MABs in boys with DMD in a first-in-human phase 1/2 clinical study demonstrated that the treatment was relatively safe and that dystrophin-positive fibers were detected but not sufficient for functional improvement. As MABs are not immune privileged, usage of immunosuppressive agents was required upon transplantation of allogeneic MABs, which might have a negative effect on homing of the MABs.^13^ The use of autologous MABs eliminates the use of immunosuppressive agents but requires the availability of MABs with no or a low mutation load. We previously demonstrated for heteroplasmic m.3243A>G mutation that six out of nine MABs cultures displayed a mutation load below 15%,^14^ enabling direct *ex vivo* expansion of patient-derived healthy MABs for autologous cell therapy to combat MMs.

Here, we present our first-in-human phase 1/2 clinical trial of autologous MABs as ATMPs. The primary goal was to assess the safety of one administration of 50 million/kg autologous MABs in the tibial artery of three patients heteroplasmic for the m.3243A>G mutation (ClinicalTrials.gov: NCT05063721). Since carriers of mtDNA mutations typically do not experience muscle wasting and associated regeneration, as seen in DMD, and MABs require muscle damage and inflammation for extravasation,^10^^,^^15^ a bout of eccentric exercise was performed 24 h prior to transplantation. This eccentric exercise induces muscle damage, infiltration of leukocytes, and expression of inflammation markers.^16^^,^^17^ Following administration of the MABs, participants were observed in the hospital for 24 h post-infusion and (serious) adverse events (SAEs) were documented. Pre- and post-infusion angiographies were performed. Plasma creatine kinase (CK), CK-MB, and lactate dehydrogenase (LDH) were analyzed as markers of (skeletal muscle) tissue damage. Plasma and tibialis anterior muscle inflammation was assessed using the pleiotropic cytokine interleukin 6 (IL6), which is a key mediator in acute-phase response to infections and injuries; the early-response cytokine tumor necrosis factor (TNF), which is mostly involved in localized tissue inflammation; and C-X-C motif chemokine ligand 12 (CXCL12) as it is a highly effective chemoattractant that activates leukocytes and is induced by pro-inflammatory cytokines, such as TNF.

Our secondary goal was to assess preliminary effectiveness with respect to skeletal muscle homing and regeneration. MABs can be discriminated in a muscle biopsy by the expression of pericyte markers, such as alkaline phosphatase and neural glia antigen-2 (NG2).^12^ However, upon autologous transplantation, the transplanted cells cannot be discriminated from the residing MABs. Indocyanine green (ICG) dye is aUS Food and Drug Administration (FDA)-approved dye that is widely used *in vivo* with near-infrared light.^18^ In this study, ICG labeling of MABs prior to infusion was tested to enable migration assessment of infused MABs in tibialis anterior muscle biopsies of treated and untreated legs 24 h after administration. For reasons explained in the results, this was done in only 1 patient. In addition, neural cell adhesion molecule (NCAM) staining was used to quantify the formation of new muscle fibers within these muscles 4–6 weeks after ATMP administration.

## Results

### Study population

From November 2020 to May 2022, six m.3243A>G mutation carriers were enrolled in this study and completed the screening visit (visit 1), which consisted of a clinical and neurological examination, checking inclusion and exclusion criteria, describing medical history, and obtaining a skeletal muscle biopsy from the m. vastus lateralis for analysis of the m.3243A>G mutation load in MABs. Five out of six participants were eligible for participation based on their m.3243A>G mutation load in MABs being <15% (Table 1). Patient D displayed an m.3243A>G mutation load of 19% in MABs, and therefore her study participation ended after visit 1. Participant E was excluded from further participation because of a vascular obstruction in his left leg, and participant F was excluded based on the investigator’s opinion with respect to his ability to understand and follow the study procedures. Participants A, B, and C were included and completed the clinical study (visits 2–5).

**Table 1:** Subjects included in phase 1/2 clinical study

ID	Age, y	Gender	Weight, kg	m.3243A>G % skeletal muscle^a^	m.3243A>G % MABs^b^	MRC sum score^c^	Clinical phenotype	Status
A	52	F	70	63.1	6.7	110	diabetes mellitus	completed all 5 study visits
B	33	F	58	55.0	7.5	110	myalgia after exercise	completed all 5 study visits
C	53	M	68	58.8	9.5	106	diabetes mellitus, hypertension, small-fiber neuropathy; weakness hip flexors and knee flexors; MRC score 4	completed all 5 study visits
D	44	F	60	68.0	19	110	hypertension; exercise intolerance	excluded after visit 1 based on mutation load in MABs >15%
E	67	M	73	65.0	6.5	110	Stenosis left common iliac artery and superficial femoral artery	excluded after visit 1 due to vascular obstruction in left leg
F	35	M	55	57.0	8.6	110	diabetes mellitus	excluded after visit 1 based on opinion of investigators

a Vastus lateralis skeletal muscle biopsy.

b Analyzed at visit 1.

c MRC sum score; maximum score is 110.

### Autologous MAB medicinal cell products

In the Good Manufacturing Practice (GMP) facility of the Leiden University Medical Center (LUMC), MABs were isolated and expanded from m. vastus lateralis biopsies taken from subjects A, B, and C for the preparation of the MP for infusion. After isolation and expansion, a batch of MABs was frozen and analysis of the intermediate product release criteria was performed. All intermediate product criteria were met—only the MAB m.3243A>G mutation load of subject C was out of specification, namely 16%, while the criterion was ≤15%. With the consent of participant C, intermediate product was used for the generation of the MP for clinical administration. One week before the scheduled date of intra-arterial transplantation, the MAB intermediate product was thawed and expanded. To enable detection of the transplanted MABs in the muscle biopsy collected 24 h after ATMP transplantation, 10% of the autologous MABs of participant B were labeled with ICG before harvesting. In this case, the ATMP consisted of 148 million (90%) unlabeled MABs and 14.8 million (10%) ICG-labeled MABs in 29 mL hypothermosol (5 million/mL) and 5 U/mL heparin. As explained in the results section, for participants A and C, the MP consisted of 175 and 168 million, respectively, unlabeled autologous MABs in 35 and 34 mL hypothermosol (5 million/mL) and 5 U/mL heparin. All three MPs fulfilled conditional release criteria (Table S1A), were Qualified Person released at the GMP facility, then transported to Maastricht University Medical Centre+ (MUMC+) and stored both at 2°C–8°C until administration. All three administrations took place within the allotted 30-h window after formulation. The final MP release criteria that only became available after administration consisted of mycoplasma, final microbiological control of cellular product, endotoxin, patient verification, and m.3243A>G mutation load analysis (Table S1B). Except for m.3243A>G mutation load analysis, all were within specifications. The m.3243A>G mutation load criterion was set at ≤15%, but were 18%, 27%, and 23% for subjects A, B and C, respectively. Participants A and C showed on average a 7%–8% increase between intermediate product (IP) and mean MP m.3243A>G mutation load analysis. The increase in participant B in the clinical study was 15%, resulting in a mutation load of 27% in the MP. In addition, a large variation (mean difference 6%) was observed upon analysis of MP mutation load using two different methods, namely NGS PacBio analysis and GeneScan fragment analysis (Table S4).

### Primary objective: Safety of intra-arterial administration of autologous MABs

**Figure 1:**
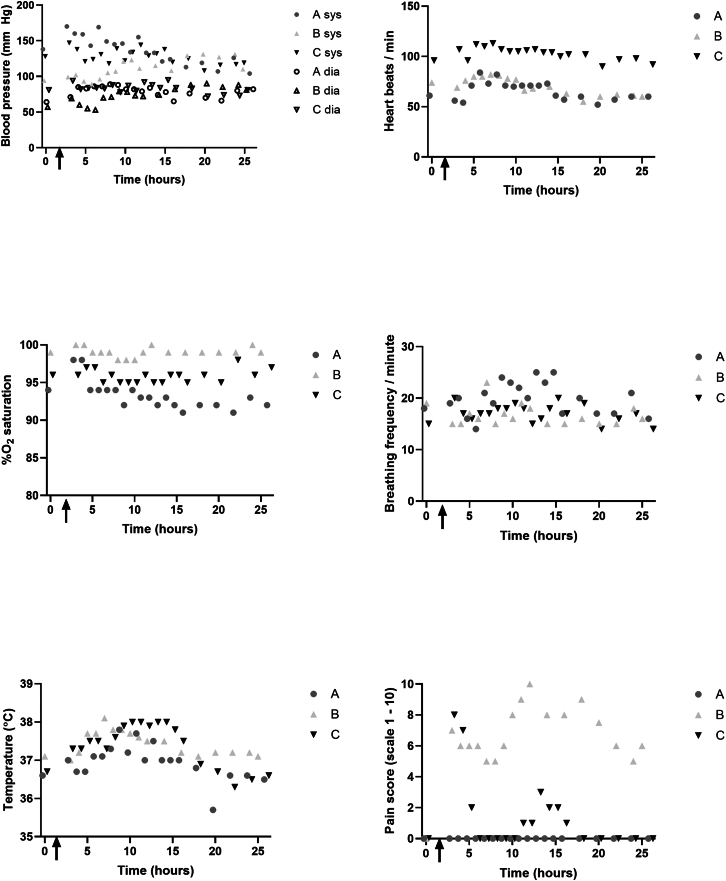
Monitoring of vital signs during 24 h after ATMP delivery At visit 4, blood pressure, heart rate, %O_2_ saturation and breathing frequency, temperature, and pain score were assessed before (t = 0) and every hour following the ATMP delivery for a total of 24 h. Symbols correspond to the subject ID: subject A: dark grey circles; subject B: light grey triangles; subject C: black inverted triangles. The arrow indicates timepoint of ATMP delivery.

As this was the first-in-human clinical administration of autologous MABs, safety assessment was the primary study objective. Pre- and post-administration angiography verified that there were no vascular obstructions in the left lower leg following autologous MAB administration. In none of the three participants were drastic changes or values outside the normal range observed of blood pressure, heart rate, breathing frequency, and oxygen saturation during 24 h of monitoring following transplantation (Figure 1; Table 2). For all participants, an electrocardiogram before and 24 h after ATMP administration demonstrated no changes for the heart.

**Table 2:** Summary of AEs, SAEs, and vital signs during 24-h monitoring following MAB administration

ID	SAE	AE	BP (mm Hg)		HR/min	%O_2_	BF/min
			SYS	DIA			
A	no	no	137 ± 20	79 ± 8	66 ± 9	93 ± 2	20 ± 3
B	no	grade II, rash, and redness TA muscle left leg	111 ± 14	74 ± 9	70 ± 9	99 ± 1	17 ± 2
C	no	no	128 ± 10	83 ± 6	103 ± 6	96 ± 1	17 ± 2

BP, HR/min, %O_2_, and BF are shown as mean ± SD during 24-h observation. AE, adverse event; BF/min, breathing frequency per minute; BP, blood pressure; DIA, diastolic blood pressure; HR/min, heart rate/minute; %O_2_, percentage O_2_ saturation; SAE, serious adverse event; SYS, systolic blood pressure.

All three subjects completed the five study visits, and no SAE or AE was observed between visits 2 and 5 in the 28 days following ATMP delivery. In subject B, a local grade II AE was observed during the ATMP administration. This local AE started as a local skin rash with itching, and redness of the skin on the front side of the left lower leg; this was in hours after the procedure followed by soreness and mild swelling of the m. tibialis anterior. Participant B was discharged as scheduled after 24-h monitoring, receiving ibuprofen (400 mg) pain medication, and an extra visit was scheduled for inspection of the leg. No 24-h muscle biopsies were taken. When the participant visited the outpatient clinic 3 days later, the rash had largely disappeared, redness of the skin was reduced, and the soreness was less. Participant B received 90% unlabeled and 10% ICG-labeled MABs as ATMP. As it was not possible to assess whether the AE was due to the use of ICG or due to a sensitivity of the patient to other components of the ATMP or the administration procedure, we decided to leave out ICG in subsequent ATMPs to eliminate a variable for safety as primary endpoint, which will not be present in the eventual therapeutic procedure. In both participants A and C, intra-arterial administration of the unlabeled MABs as ATMPs was generally well tolerated. Participant A experienced discomfort in the treated left lower leg during administration, which improved by moving her feet, and the discomfort completely resolved after ATMP administration was completed. Participant C experienced pain in the left leg toward the end of the intra-arterial ATMP administration and during the 10-min pressure on thigh by the intervention radiologist upon removing the catheter from the femoral artery. Paracetamol was given, and the pain was nearly gone within a couple of hours after the procedure. The front side of the treated m. tibialis anterior was a bit warm in participants A and C and also a bit red in participant C, during the first hours after the procedure.

**Figure 2:**
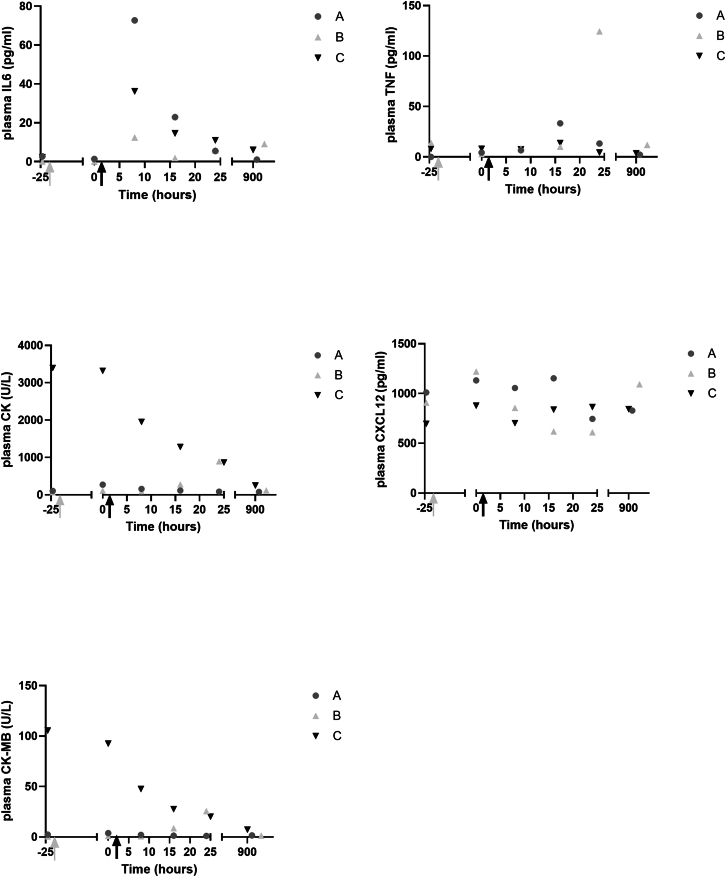
Assessment of body temperature, pain score, and markers of inflammation and tissue damage in venous blood samples following ATMP delivery ATMP administration was performed at t = 1–2 h, venous blood samples were collected before ATMP administration at visit 3 (t = −24 h) and at start of visit 4 (t = 0 h), three times after ATMP delivery at visit 4 (t = 8, 16, and 24 h), and at visit 5 (4–6 weeks after ATMP delivery). The samples were analyzed for IL6, TNF, CK, CXCL12, and CK-MB. The gray arrow indicates time point eccentric exercise bout with lower legs at visit 3, and the black arrow indicates time point ATMP infusion at visit 4. Symbols correspond to the subject ID: subject A: dark grey circles; subject B: light grey triangles; subject C: black inverted triangles.

To assess systemic inflammation due to ATMP delivery, multiple blood samples were collected, two of which occurred before ATMP administration (time [t] = −24 h and t = 0 h), and four after administration (t = 8, 16, and 24 h and 4–6 weeks). In these blood samples, IL6, CK, CK-MB, LDH, TNF, and CXCL12 were analyzed (Figure 2). The IL6 plasma level was elevated at blood sampling 8 h after administration but returned to baseline within 24 h after ATMP administration. This ceased within a couple of hours after administration, when administered cells were cleared from the circulation, causing cessation of the IL6 signaling cascade. In line with this, LDH, CK, and CK-MB were not elevated in participants A and C. Only in participant B, who experienced the AE, were CK and CK-MB increased 24 h after ATMP administration, which was back to normal at the last study visit. The same was observed for TNF. Lastly, ATMP administration did not impact plasma CXCL12 levels. Lack of CXCL12 activation is in line with the other parameters, verifying that autologous MABs delivery as ATMP is not associated with a systemic inflammation or an immune response.

**Figure 3:**
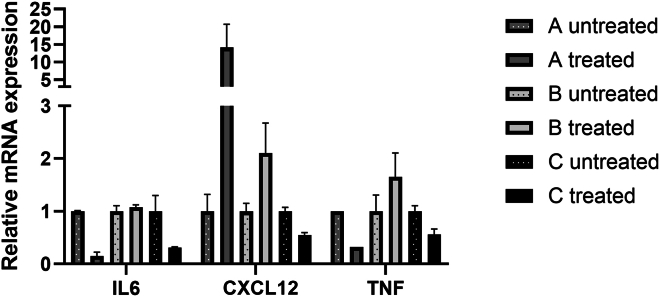
Assessment of skeletal muscle inflammation following ATMP delivery Relative mRNA expression in treated and untreated tibialis anterior muscle biopsy at visit 5 of IL6, CXCL12 and TNF was normalized to TATA-box-binding protein housekeeping gene. Relative gene expression of the treated sample was compared with individual’s untreated sample; data is shown as mean ± SD, a >2-fold change is considered increased. One muscle sample was collected from left and right m. tibialis anterior from each participant for RNA isolation and cDNA isolation. Quantitative PCR was performed in triplicate.

To assess whether autologous MAB administration resulted in inflammation of the m. tibialis anterior, H&E staining and gene expression analysis of inflammation markers TNF, IL6, and CXCL12 was performed. Data of treated tibialis anterior muscle were compared with untreated tibialis anterior of each individual (Figure 3). A 2-fold increase in gene expression was considered to be upregulated expression. Gene expression of IL6 and TNF was in none of the three participants increased >2-fold due to MAB delivery. Only expression of chemokine CXCL12 was increased >2-fold in the treated muscles of participants A and B. In addition, analysis of H&E-stained muscle samples by a pathologist verified that MAB administration did not result in an increased presence of leukocytes in the MAB-treated leg. In summary, autologous MAB administration does not result in a skeletal muscle inflammation response.

### Secondary objectives: Skeletal muscle homing and muscle regeneration

**Figure 4:**
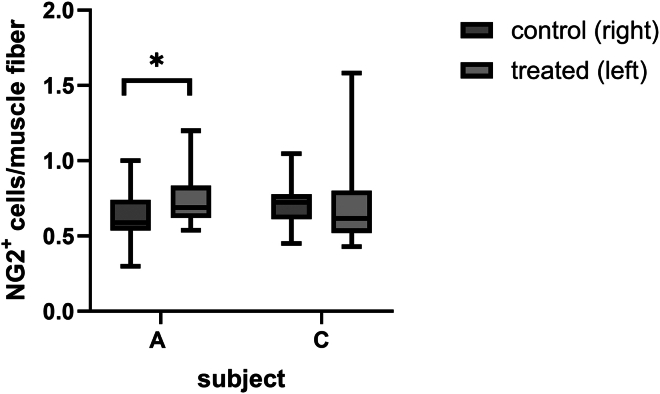
Quantification of NG2^+^ cells/muscle fiber At 24 h after intra-arterial MAB administration in left lower leg in the phase 1 study, m. tibialis anterior biopsies of the left (treated) and right (untreated) legs were collected from two out of the three subjects (A and C). Per biopsy, a minimum of 400 muscle fibers were imaged for WGA to quantify the number of muscle fibers/field and NG2^+^ to count the number of MABs. Boxes represent the interquartile range (25th to 75th percentile), the line within the box indicates the median, and whiskers extend to the minimum and maximum values, ∗*p* < 0,05 Counting was performed blinded.

**Figure 5:**
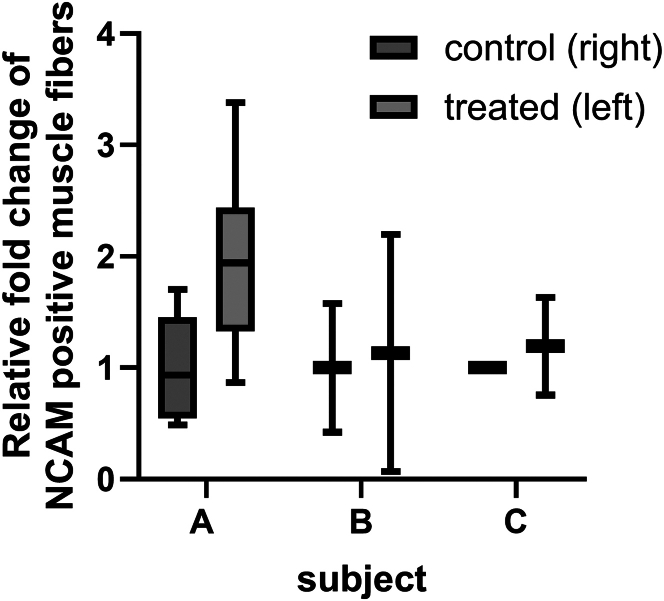
NCAM staining of tibialis anterior muscle biopsy of treated and untreated legs Following DAB-NCAM staining, quantification of NCAM^+^ and NCAM^−^ muscle fibers. Per subject, two muscle biopsies of left and right m. tibialis anterior were analyzed. Boxes represent the interquartile range (25th to 75th percentile), the line within the box indicates the median, and whiskers extend to the minimum and maximum values. For subject C, only one muscle sample of the right tibialis anterior (control) was available for quantification. A minimum of 300 muscle fibers was quantified per sample by a blinded researcher.

The secondary objective of the phase 1/2 clinical trial was to assess MAB homing and induction of muscle regeneration in m. tibialis anterior of the treated leg. To stimulate homing, 1 min 40 s bouts of dorsiflexion exercise were performed with both legs until exhaustion on a Biodex device. As shown in Figure S1, a 36%–42% reduction in the cumulative amount of physical work performed (total work) of the left leg was observed in the last set compared to the first set of subjects A and C. Subject B did not show a difference in total work (Joules [J]) between first and last exercise bout, even though she performed more bouts than subjects A and C. Assessment of migration of ICG-labeled MABs was not possible, as ICG labeling was discontinued after an AE in participant B, and no muscle biopsies were taken due to the discomfort. Alternatively, we assessed MAB migration by quantification of the total number of NG2^+^ cells, as markers for both residing and administered MABs, per muscle fiber in biopsies of the treated and untreated m. tibialis anterior 24 h after ATMP administration in participants A and C. As shown in Figure 4, a significant increase of 20% ± 7% (*p* < 0.05) in NG2^+^ cells/muscle fiber was observed in a biopsy of the treated muscle versus the untreated muscle in participant A. In participant C, no significant increase was observed, but fields with increased numbers of NG2^+^cells/fiber were observed in muscles of the MABs-treated leg. Microscopic analysis of the muscle biopsies collected of both treated and untreated m. tibialis anterior 4–6 weeks after MABs administration of all three subjects showed that the mean number of NCAM^+^ muscle fibers was increased in all three subjects, but a large variation exists between regions in the muscle biopsies (Figure 5).

## Discussion

Here, we report the results of the first phase 1/2 clinical trial of human intra-arterial administration of autologous MABs in the m. tibialis anterior of m.3243A>G mutation carriers to assess primarily safety and secondary efficacy with respect to homing and induction of muscle regeneration. We found that autologous MAB transplantation in tibial artery is safe and well tolerated. Comparison of the muscle biopsy of the treated versus the untreated contralateral m. tibialis anterior suggests homing and regeneration of administered MABs.

### ATMP production

A total of 50 million MABs/kg body weight of transplanted limb (lower leg 5% of body weight) were transplanted as used by Cossu et al. with allogeneic MABs.^13^ For intra-arterial transplantation of one m. tibialis anterior, sufficient autologous MABs could be cultured in the GMP facility from all three m.3243A>G mutation carriers within six to seven passages, demonstrating the ability to culture 3.8 billion MABs for future transplantation of all muscle groups in the body within 15 passages, thus preserving the capacity of MABs to proliferate *in vivo* following transplantation.^12^^,^^14^ As autologous MABs were used, the m.3243A>G mutation load was determined in the IP after magnetic-activated cell sorting (MACS) at passage 3 and in the MP at passage 6/7. During MABs expansion, the m.3243A>G mutation load increased on average 7%–8%, and in one case, 15%. This does not lead to safety issues as the likelihood of being unaffected at 27% is around 90%,^19^ and the mutation load is still >25% below the skeletal muscle mutation load of the individual. This increase in mutation load was not observed when MABs from these participants, collected at another study visit, were cultured under the same conditions in a research lab, nor was this observed in MABs from four additional m.3243A>G mutation carriers (see Figure S3). Changes in the mtDNA mutation load have been reported to occur during culture of primary cells or mtDNA mutation carrier,^20^^,^^21^ which may be random and cannot be prevented. Based on these limited data, it is not possible to draw conclusions on whether or not this is a consistent phenomenon in a GMP culture or a coincidence. In addition, the variation between replicates and methods for m.3243A>G mutation load analysis needs to be reduced.

### Safety

Our primary aim was to show the safety of intra-arterially injecting ∼250 million autologous MABs in the tibial artery. No SAE, no vascular obstructions measured by angiography, and no changes in heart rate, blood pressure, oxygen saturation, or breathing frequency were observed during 24 h following MAB administration. One grade II local AE was observed and resulted in elevated pain score, namely local inflammation in the lower leg of participant B treated with 10% ICG-labeled MABs in hypothermosol to enable the quantification of autologous ICG^+^ MABs in the m. tibialis anterior biopsy. We consider it unlikely that this local AE is caused by the MABs as they were patient own, displayed a high viability, and were checked for contaminants and aggregates, and the excipient solution hypothermosol is FDA approved and has been used in many clinical studies using various administration routes. ICG binds to plasma proteins and is among the others used for measuring cardiac output and to visualize blood vessels and lymph nodes during surgeries. Reported AEs of ICG include urticarial reactions,^22^ which were observed in participant B. Since it is not possible to discriminate whether the AE is caused by the ICG or by the sensitivity of participant B to (other components of) the MAB administration, we decided to discontinue the ICG, and participants A and C were treated with unlabeled MABs. In contrast to the inflammation of the treated left lower leg of patient B, the front side of the treated m. tibialis anterior felt a bit warm in participants A and C, and a temporal increase in plasma IL6 and TNF at 8 and 16 h post-administration of all participants may be responsible for this slight elevation of 1.17°C ± 0.15°C in body temperature. The direct and short window of increased IL6 is likely linked to cellular stress due to the ATMP delivery procedure. In contrast, CXCL12 was not increased in plasma following intra-arterial transplantation, but it was increased in skeletal muscle biopsy of treated m. tibialis anterior 4–6 weeks after MAB administration in participants A and B. CXCL12 expression is associated with increased muscle regeneration,^23^ but it would require further investigation to show that this is the cause in these participants.

No immune response was observed in the present study. This was expected as autologous MABs were transplanted and since wild-type tRNAleu is present in all patients with the heteroplasmic m.3243A>G mutation. This suggests that mtDNA mutation carriers are more suitable candidates for cell therapy compared to carriers of a nuclear DNA mutation(s) that completely lack the wild-type protein. For example, antibodies against wild-type dystrophin protein have been observed upon transplantation in patients with DMD.^24^ The intra-arterial administration of autologous MABs as ATMP is safe, but all patients experienced some discomfort, which manifested from very mild (patient A) to more severe (patient B), which could be an individual-specific reaction to the ATMP and procedure or related to ICG. Since this is only the second clinical study where MABs were administered intra-arterially and the first study using autologous MABs, further research is needed to determine the cause of the discomfort. In a previous DMD trial, donor MABs were administered under general anesthesia,^12^ making it unclear whether the discomfort observed is specific to autologous MAB administration, MAB administration in general, or related to the ATMP administration procedure itself. For example, it is worth investigating whether adjusting the procedure, such as administering the ATMP at a slower rate or pre-warming it to 37°C, might reduce the discomfort.

### Homing and regeneration capacity

The secondary objective was to assess the migration and regenerative capacity of autologous MABs upon intra-arterial delivery. Muscle damage and the resulting inflammation is required for MAB migration from the artery to the muscle^10^ and can be present due to the disease (e.g., in MDs) or can be triggered by inducing muscle damage (e.g., cardiotoxin injection in mice).^25^ In humans, eccentric exercise induces muscle damage, which can be measured by the expression of infiltration markers. Also, Paulsen et al. demonstrated inflammation and leukocyte infiltration in exercised muscles following exercise-induced 40%–50% reduction of maximum strength.^16^ As muscle damage is generally not present in m.3243A>G carriers, including the subjects in this study, dorsiflexion exercise sessions to exhaustion were performed with both lower legs 24 h before intra-arterial MAB administration. Although we did not quantify peak torque before and after the eccentric exercise, on average, total work (J) was reduced by 26% ± 8% (mean ± SEM) in the last session of 1 min 40 s compared to the first session, it is reasonable to expect that muscle damage and local inflammation was induced in our study. To assess MABs homing, quantification of NG2^+^ cells in muscle biopsies was collected 24 h after administration, which has been described in the literature for MAB quantification.^26^^,^^27^ Quantification of NG2^+^ cells per muscle fiber showed a significantly higher number of MABs in the transplanted m. tibialis anterior of individual A, while individual C showed some fields with increased NG2^+^ cells, but this was not significantly different from the untreated m. tibialis anterior. These data suggest homing of intra-arterial-transplanted autologous MABs, but without labeling we cannot discriminate between autologous-transplanted and residing MABs. Torrente et al. quantified intra-muscularly injected autologous CD133^+^ cells in muscle cryosections using Prussian blue staining of iron dyne beads that were incorporated upon CD133^+^ MACS sorting.^24^ This approach was successful for assessing intra-muscularly injected cells, but it requires the availability of GMP-grade antibody-coated magnetic beads. In addition, a biopsy may not represent the entire muscle upon intra-arterial administration of stem cells. To evaluate the homing of systemically administered stem cells and assess their distribution overall in muscle groups, it is essential to develop GMP-compliant cell-labeling procedures that provide sufficient signal intensity for non-invasive detection methods.

### Conclusion

This phase 1/2 clinical study demonstrated that intra-arterial delivery of autologous MABs as ATMPs is safe and well tolerated. Data with respect to homing of the MABs to the transplanted muscle were promising but not conclusive. Based on animal studies, a minimum of three MAB doses at 4- to 6-week interval is needed to achieve functional improvement. This will be the aim of the next phase 2a intra-subject controlled study (NCT05962333).

## Materials and methods

### Study design

**Figure 6:**
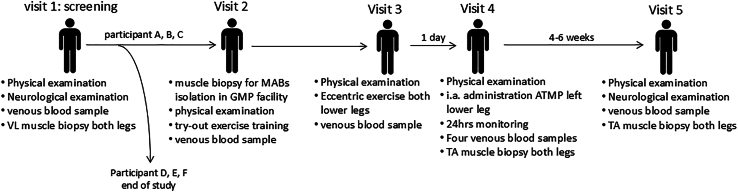
Study design Study design, including interventions at each study visit. i.a., intra-arterial; TA, tibialis anterior; VL, vastus lateralis.

This intra-subject controlled mono-center study, approved by the Central Committee on Research Involving Human Subjects in the Netherlands (NL68732.000.19, June 7, 2020), was conducted according to the principles of the Declaration of Helsinki and in accordance with the Medical Research Involving Human Subjects Act and other guidelines, regulations, and acts. Carriers of the m.3243A>G mutation were enrolled in this phase 1/2 clinical study at MUMC+ in the Netherlands, and written informed consent was obtained before execution of the study. This study comprised five study visits (see Figure 6 for an overview of the study design). Visit one was a screening visit to assess eligibility for participation. At this visit, inclusion and exclusion criteria, virology (HIV/hepatitis/human T lymphotropic virus [HTLV]/*Treponema pallidum* hemagglutination [TPHA]), and medical history were evaluated, a neurological and physical examination was performed, and a skeletal muscle biopsy was obtained from the m. vastus lateralis to determine whether the m.3243A>G mutation load in MABs was below 15%. At visit 2, a skeletal muscle biopsy from the m. vastus lateralis was collected from eligible participants for isolation and expansion of autologous MABs at the GMP facility of the LUMC. Upon completion of MABs expansion, visits 3 and 4 were scheduled. At visit 3, 1 day before transplantation, a maximal bout of eccentric exercise was performed with both lower legs to induce muscle damage and inflammation. The next day (visit 4), autologous MABs were administered into the tibialis anterior of the left leg via a catheter in the femoral artery. Pre- and post-administration angiography was performed, and the participants were observed for 24 h, vital signs were recorded (bi)hourly, and venous blood samples were collected every 8 h. At 24 h after transplantation, an m. tibialis anterior biopsy of both the left (treated) and right (untreated) legs was collected. Patients returned to the hospital 4–6 weeks later for the last study visit (visit 5). At this study visit, a physical and neurological examination was performed, a venous blood sample was collected, and an m. tibialis anterior biopsy of both the left (treated) and right (untreated) legs was collected. The following paragraphs describe all procedures used.

### Clinical and neurological examination

A baseline routine clinical examination was performed at each study visit to record the body height and weight, the blood pressure, and the heart rate at rest. A neurological clinical examination was performed at the first and last visits to document the disease course and included interval medical history, present illness, and general medical history; eye motor and verbal (EMV) response score; global mental status; cranial nerves; muscle strength (Medical Research Council [MRC] sum score) muscle tone, and muscle bulk; gait testing; sensory and coordination examination; and reflex testing.

### Skeletal muscle biopsies

The m. vastus lateralis and tibialis anterior skeletal muscle biopsies were collected using the 14G Pro-Mag I automatic biopsy instrument. First, the skin was locally anesthetized using 2–3 mL lidocaine, and a 0.5-cm incision was made in skin and fascia, after which the muscle samples were collected using the Pro-Mag I automatic biopsy instrument. Following the muscle biopsy, the skin was closed using a Steristrip and covered by a Tegaderm. The m. vastus lateralis samples (∼100 mg) collected for MAB culture were transferred sterile into a 50-mL tube containing 20 mL Iscove’s modified Dulbecco’s medium (IMDM) medium supplemented with 50 μg/mL gentamycin, 1% insulin-transferrin-selenium, 1% non-essential amino acids, 0.1 mM 2-mercaptoethanol, and 10 ng/mL fibroblast growth factor (FGF). The tibialis anterior biopsies were embedded in Tissue-Tek and subsequently snap-frozen in liquid nitrogen-cooled isopentane.

### MAB isolation, expansion, ICG labeling, and MP formulation for clinical administration

The sterile collected muscle tissue, the starting material of the production process, was transported cooled (2°C–8°C) to the GMP facility. Following reading of the temperature logger, checking of the transport documents, and initiation of sterility tests, the start material was dissected into small pieces (±2 mm) using a surgical knife, and fat was removed when present. Selected fragments were transferred into type I human collagen (Symatese)-coated 10-cm dishes containing 4 mL complete MABs medium (IMDM supplemented with 10% fetal bovine serum (FBS), 50 μg/mL gentamycin, 1% insulin-transferrin-selenium, 1% non-essential amino acids, 0.1 mM 2-mercaptoethanol, and 10 ng/mL FGF) and were incubated at 37°C/4% O_2_/5% CO_2_. After ∼1 week, the preliminary growth of adherent cells became visible. A characteristic of pericytes is that they are initially apparent as small, round, very refractile cells that are floating or weakly adherent to the layer of flat cells below. After ∼2 weeks, the culture medium and floating fragments (cells/pieces of muscle tissue) were carefully discarded, the adherent cells were detached using TrypLE Select and reseeded at 10,000–12,000 cells/cm^2^ in MAB medium in a new flask. At 1 and 2 days later, the floating cells (pericyte-derived progeny: MABs) were recovered and transferred to new flasks at a density of 4,000–6,000 cells/cm^2^. MABs were expanded, CD56^+^ cells were depleted using CliniMACS CD56 reagent (Miltenyi Biotec), and the IP was frozen in 90% FBS/10% DMSO at a concentration of 1 × 10E−6/mL. IP testing comprised immunophenotyping (CD13^+^, CD44^+^, CD31^−^, CD34^−^, CD45^−^, and CD56^−^) using an FACS Canto II (BD). Viability and cell number quantification were obtained using trypan blue staining and hemacytometer counting. m.3243A>G mutation load analysis was performed as described below. Patient identity verification was checked using the AmpFLSTR kit and limulus amebocyte lysate assay for endotoxin. Mycoplasma PCR test and Bactec test for microbiological control of cellular product were performed by the Department of Clinical Microbiology at LUMC. At 1–2 weeks before infusion, IP was thawed, expanded for 1–2 passages, and MP was formulated 1 day before infusion. The MP consisted of either unlabeled or 90% unlabeled and 10% labeled with ICG (Verdye) at a concentration of 30 μg/mL in culture medium for a minimum of 1 h and a maximum of 3 h before harvesting. After harvesting, the autologous MABs were washed with NaCl with albumin, and the required number of MABs (50 million × 5% of body weight for treatment of one lower leg) was re-suspended in hypothermosol (BioLife Solutions) and 5 IU/mL heparin (5 × 10E−6/mL). The MP was stored and transported to MUMC+ between 2°C and 8°C until infusion.

### Quantification of m.3243A>G mutation load

For quantification of the m.3243A>G mutation load in MABs and skeletal muscle, DNA was isolated using the Promega Wizard DNA extraction kit according to the manufacturer’s instructions. Quantification of the m.3243A>G mutation load was performed using GeneScan fragment analysis of *HaeIII*-digested PCR products, which results in a 48-bp fragment in the case of an m.3243A>G mutation and a 122-bp fragment for wild-type mtDNA, as described.^14^ Alternatively, next-generation sequencing of m.3243-containing PCR product was performed using a PacBio analyzer at the Department of Clinical Genetics of MUMC+.

### Eccentric exercise of m. tibialis anterior

A bout of eccentric exercise training of both lower legs was performed on a Biodex isokinetic dynamometer to induce muscle damage and inflammation of the m. tibialis anterior. During exercise training, patients were positioned in the supine lying position, where resistance was applied to the dorsum of the foot just above the toes to resist dorsiflexion, while stabilization was applied to the lower leg. The amount of resistance increased gradually to be suitable to the participant’s tolerance. Each subject rested for 30 s after each set of 10 contractions to avoid fatigue. Participants performed as many sets of 10 contractions as feasible for them.

### Intra-arterial administration of MP, angiography, and 24-h monitoring

The MP was intra-arterially delivered to the anterior tibial artery (ATA) of the left lower leg via a catheter in the left femoral artery. Following local anesthesia with 10 mL lidocaine 1%, a 4-Fr introducer sheath was inserted antegrade in the common femoral artery followed by an injection of 3,000 U heparin through the sheath. Initial digital subtraction angiography (DSA) targeted on the vessels of the lower leg (6 mL of 50% diluted contrast agent with a 3-mL/s injection rate) was performed. The ATA was identified and selectively catheterized with a 2.7-Fr micro-catheter (Progreat Terumo) using a micro-guidewire (0.021 in). Then, the ATMP was injected at a rate of 1–2 mL/min in the target vessel under continuous fluoroscopy. After finishing the procedure, post-injection DSA was acquired to assess patency of the culprit vessels. The sheath was removed, and the puncture location was manually compressed for at least 10 min. Subsequently, the patient was immobilized for at least 4 h and monitored for 24 h in the hospital after infusion. Each hour, the participant’s temperature, blood pressure, heart rate, oxygen saturation, and breathing frequency per minute were recorded, as well as pain score (0 being no pain and 10 being very much pain), EMV score (maximal score 4-6-5), MRC strength score of both legs (0–5), and visual inspection of the legs.

### Venous blood sampling and analysis

At study visits 1, 2, 3, and 5, one venous blood sample was collected and four samples every 8 h were collected during 24-h monitoring at visit 4. Upon collection of venous blood, samples were centrifuged (20 min at 2,000 rpm), and plasma was either analyzed directly or stored in cryovials at −80°C until analysis. Analysis of creatinine, CK, CK-MB, and lactate were performed by the MUMC Central Diagnostics Laboratory. Analysis of hepatitis B/C, HIV, HTLV, and TPHA were performed by the MUMC Department of Microbiology and Virology. Plasma levels of TNF, CXCL12, and IL6 were analyzed using ELISA according to the manufacturer’s protocol (R&D Systems), and signal intensity was assessed using a CLARIOstar microplate reader (BMG Labtech).

### (Immuno)stainings of skeletal muscle biopsies

Skeletal muscle biopsies of m. tibialis anterior were snap-frozen upon collection in O.C.T. (TissueTek) in isopentane cooled using liquid nitrogen and stored at −80°C. Muscle cross-sections of 7 μm were made using a cryotome (Leica) and directly frozen at −20°C. Before immunostaining, the cryosections were fixated with 4% paraformaldehyde (Sigma-Aldrich) for 10 min, followed by washing three times with PBS. To assess homing in biopsies collected 24 h after administration, cryosections were incubated for 90 min in blocking solution (PBS containing 2% bovine serum albumin, 5% FBS, 0.2% Triton X-100, and 0.1% sodium azide), washed three times with PBS, and incubated overnight with primary antibody anti-NG2 (MAB5384-1, Millipore Sigma) in a blocking solution at 4°C. After overnight incubation, the cryosection was washed three times with PBS and incubated with 1:500 Alexa Fluor 594 goat anti-mouse secondary antibody and 1:500 wheat germ agglutinin (WGA) 488 goat anti-rabbit (ab73593, Abcam) for 1 h at room temperature. Nuclei were stained with DAPI in glycerol. Slides were imaged on an Olympus BX51 fluorescent microscope. Images of NG2 and WGA staining were merged using ImageJ, and the number of NG2^+^ cells and the number of muscle fibers were counted manually. For quantification, slides were randomized, and counting was performed by a blinded researcher. A minimum of six fields of view were quantified per sample. To assess the formation of new muscle fibers in muscle biopsies collected 4–6 weeks after administration, cryosections were incubated for 60 min in blocking solution (PBS containing 2% bovine serum albumin, 5% FBS, 0.2% Triton X-100, and 0.1% sodium azide), washed three times with PBS, and incubated overnight with 1:50 primary antibody anti-NCAM (5.1H11 Developmental Studies Hybridoma Bank) in a blocking solution at 4°C. After overnight incubation, the cryosection was washed three times with PBS and incubated with 1:100 rabbit anti-mouse HRP secondary antibody (Agilent DAKO) in a blocking buffer for 1 h at room temperature. After washing three times with PBS, samples were incubated with 1:50 DAB Chromogen (Agilent DAKO, K3467) in a substrate buffer for 10 min at room temperature. After washing with demiwater, nuclei were stained using hematoxylin for 2 min, washed with water for 5 min, dehydrated, and covered with a coverslip with Entellan. Samples were imaged using a 3D Histech Panoramic 1000 slide scanner. The number of NCAM+ cells and total number of muscle fibers were counted manually using 3D Histech Caseviewer software v2.4. For quantification, slides were randomized and counting was performed by a blinded researcher.

### Quantitative PCR

Total RNA was isolated from the m. tibialis anterior biopsies using TRIzol reagent, and purified with the High Pure RNA Isolation kit (Roche). cDNA was synthesized from 1 μg RNA using qSCRIPT reagent according to the manufacturer’s protocol (QuantaBio). Quantitative PCR was performed on a Roche LC480 in 10-μL reactions containing 1× SensiMix SYBR Hi-Rox (Bioline), 1.5 μM forward and reverse primer (see Table S3 for primer sequences) (IDT) and 5 ng cDNA, using the following cycling conditions: an initial step of 10 min at 95°C, followed by 40 cycles of 15 s at 95°C and 1 min at 60°C. The mRNA levels of each gene were normalized to those of the housekeeping gene TATA-box-binding protein.

### Statistical analysis

A paired Student t test was used to assess whether the number of NG2^+^ cells was significantly different between m. tibialis anterior of the treated leg compared with the untreated leg collected 24 h after transplantation and to assess significant changes in the number of NCAM^+^ muscle fibers between m. tibialis anterior of the treated leg compared with the untreated leg collected at visit 5 (4–6 weeks after transplantation) (*p* < 0.05 was considered significantly changed). For all other data, descriptive statistics were used.

## Data availability

The data that support the findings of this study are available from the corresponding author upon reasonable request.

## Acknowledgments

The authors would like to express their utmost gratitude to the m.3243A>G mutation carriers that participated in this study, without whom this study would not have been possible. We would also like to thank all monitors for observing the participants for 24 h after transplantation, the support staff from Pharmacy, Medium Care, and Interventional Radiology at MUMC, and all other persons who contributed to this study. Funding for this study was provided by 10.13039/100013276Interreg EMR116, 10.13039/100016062Metakids ING goede doelen 2014-055, the Dutch “Prinses Beatrix Spierfonds” (PBSW.0R15-09), Ride4Kids, and Join4Energy. The graphical abstract was created in BioRender (https://BioRender.com/amdxwvg).

## Author contributions

F.H.J.v.T.: study conception and design, execution and data collection, analysis and interpretation of results, manuscript preparation, and funding acquisition. J.G.J.H. and C.v.d.L.: execution and data collection and manuscript revision. C.v.d.L. and E.T.: data collection, analysis and interpretation of results, and manuscript revision. N.W.: execution and data collection and manuscript revision. P.J.L.: analysis and interpretation of results and manuscript revision. F.Y.: analysis and interpretation of results. I.M.W., S.P.M.K., P.M., and F.L.: execution and data collection and manuscript revision. H.R. and L.A.C.M.W.: execution and data collection. I.F.M.d.C.: study conception and design, execution and data collection, analysis and interpretation of results, and manuscript revision. H.J.M.S.: study conception and design, analysis and interpretation of results, manuscript revision, and funding acquisition.

## Declaration of interests

H.J.M.S. is an employee and shareholder of Milocron Therapeutics BV.

## Supplemental information

Document S1. Figures S1–S3, Tables S1–S4, and supplemental materials and methods

Document S2. Article plus supplemental information
