# Supplementary material for: Intra-arterial transplantation of autologous mesoangioblasts in m.3243A>G mutation carriers is safe: First phase 1/2 human clinical study
Source: Mol Ther. 2025 Jul 17;33(10):5061–72. doi: 10.1016/j.ymthe.2025.07.005 (PMC12848220; doi:10.1016/j.ymthe.2025.07.005)
Supplement: Document S1. Figures S1–S3, Tables S1–S4, and supplemental materials and methods [file mmc1.pdf]

YMTHE, Volume *33*

Supplemental Information

Intra-arterial transplantation of autologous mesoangioblasts in m.3243A>G mutation carriers is safe: First phase 1/2 human clinical study

Florence H.J. van Tienen, Janneke G.J. Hoeijmakers, Christiaan van der Leij, Erika Timmer, Nikki Wanders, Patrick J. Lindsey, Fangzheng Yi, Fong Lin, Susanne P.M. Kortekaas, Helene Roelofs, Inge M. Westra, Pauline Meij, Lambert A.C.M. Wijnen, Irenaeus F.M. de Coo, and Hubert J.M. Smeets

Supplemental material

In- and exclusion criteria

In order to be eligible to participate in this study, a subject must meet all of the following criteria:

- Written informed consent
- Age: 18+
- Sex: male/female
- Patients with the m.3243A>G mutation

A potential subject who meets any of the following criteria will be excluded from participation in this study:

- Use of anti-coagulants, anti-thrombotics and other medication influencing coagulation
- Have a weekly alcohol intake of ≥ 35 units (men) or ≥ 24 units (women)
- Current history of drug abuse
- Deficient immune system or autoimmune disease
- Significant concurrent illness
- Ongoing participation in other clinical trials
- Major surgery within 4 weeks of the visit
- Vaccination within 4 weeks of the visit
- Pregnant or lactating women
- Psychiatric or other disorders likely to impact on informed consent
- Patients unable and/or unwilling to comply with treatment and study instructions
- Any other factor that in the opinion of the investigator excludes the patient from the study
- A history of strokes
- Allergy for contrast fluid
- Peripheral signs of ischemia or vasculopathy

Table S1. Conditional release criteria of 3 MP batches of autologous MABs

Description		Method	Release Criteria	Subject a (MAB001 lijn 2)	Subject b (MAB002)	Subject c (MAB003)
**Cell number**		Trypan blue hemacytometer	5*10E6/ml * 40 ml**	175*10E6 in 35 ml (5*10E6/ml)	145*10E6 in 29 ml (5*10E6/ml)	170*10E6 in 34 ml (5*10E6/ml)
**Cell viability**		Trypan blue hemacytometer	>70%	88,8%	97,4%	90,4%
**Definitive result sterility start material**		Bactec	Negative	Negative	Negative	Negative
**Definitive result sterility intermediate product**		Bactec	Negative	Negative	Negative	Negative
**Definitive result sterility final product (MP)**		Bactec	Preliminary result Negative	Preliminary result Negative	Preliminary result Negative	Preliminary result Negative
**Immuno-phenotype**	**CD34**	FACS	≤ 5%	0,11%	0,14%	0,14%
	**CD31**		≤ 5%	0,2%	1,81%	0,72%
	**CD44**		≥ 90%	99,8%	99,9%	99,8%
	**CD13**		≥ 90%	99,4%	98,1%	99,3%
	**CD56**		≤ 5%	0,07%	0,54%	0,15%
	**CD45**		≤ 5%	0,12%	0,70%	0,18%

Table S2. Final release criteria of 3 MP batches of autologous MABs

Description	Method	Release Criteria	Subject a	Subject b	Subject c
**Mycoplasma test**	PCR	Negative	Negative	Negative	Negative
**Microbiological control of cellular product**	Bactec	Negative	Negative	Negative	Negative
**Endotoxin**	LAL assay	≤1 EU/ml	≤1 EU/ml	≤1 EU/ml	≤1 EU/ml
**Patient verification**	Genescan analysis	100% match	100% match	100% match	100% match
**Mean mtDNA mutation load**	Genescan analysis	<15%	18%*	27%*	23%*

* Out of specification;

Table S3. Primers qPCR

	Forward (5'-3')	Reverse (5'-3')
**TBP**	CACGAACCACGGCACTGATT	TTTTCTTGCTGCCAGTCTGGAC
**IL-6**	GGTACATCCTCGACGGCATCT	GTGCCTCTTTGCTGCTTTCAC
**SDF1a**	GTGGTCGTGCTGGTCCTC	AGATGCTTGACGTTGGCTCT
**TNFa**	GACAAGCCTGTAGCCCATGT	GAGGTACAGGCCCTCTGATG

Table S4. m.3243A>G mutation load in MABs GMP culture phase I/II clinical study

	IP Pacbio	MP Pacbio	MP genescan
**a**	6%	18%	11±1%
**b**	12%	27%	32,5±3,5%
**c**	16% 1st run<br>18% 2nd run	23%	26,5±3,5%

IP: intermediate product after MACS sorting; MP: Medicinal Product; MP2: 2nd MP sample; PacBio m.3243A>G mutation load analysis is NGS-based analysis performed by Clinical Genetics department as IP and MP release test. Genescan analysis of m.3243A>G mutation load was performed in duplicate following diagnostic SOP for m.3243A>G mutation load analysis, but performed in a research lab.

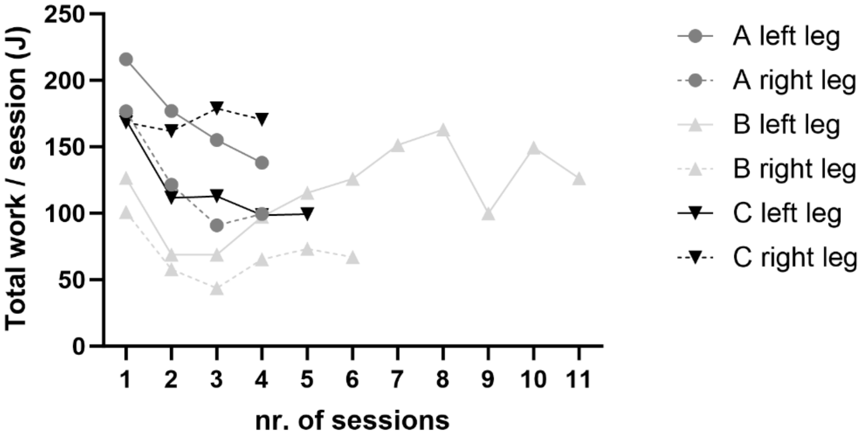

Figure S1. Eccentric exercise of lower legs 1 day before i.a. delivery

A maximum bout of eccentric exercise on a Biodex was performed with both lower legs 1 day before intra-arterial delivery of autologous MABs. Sessions of 1 minute 40 seconds were performed, followed by a 30 second break until exhaustion. Exercise was first performed with the left lower leg and the same number of sequential sessions was performed with the right leg, if possible.

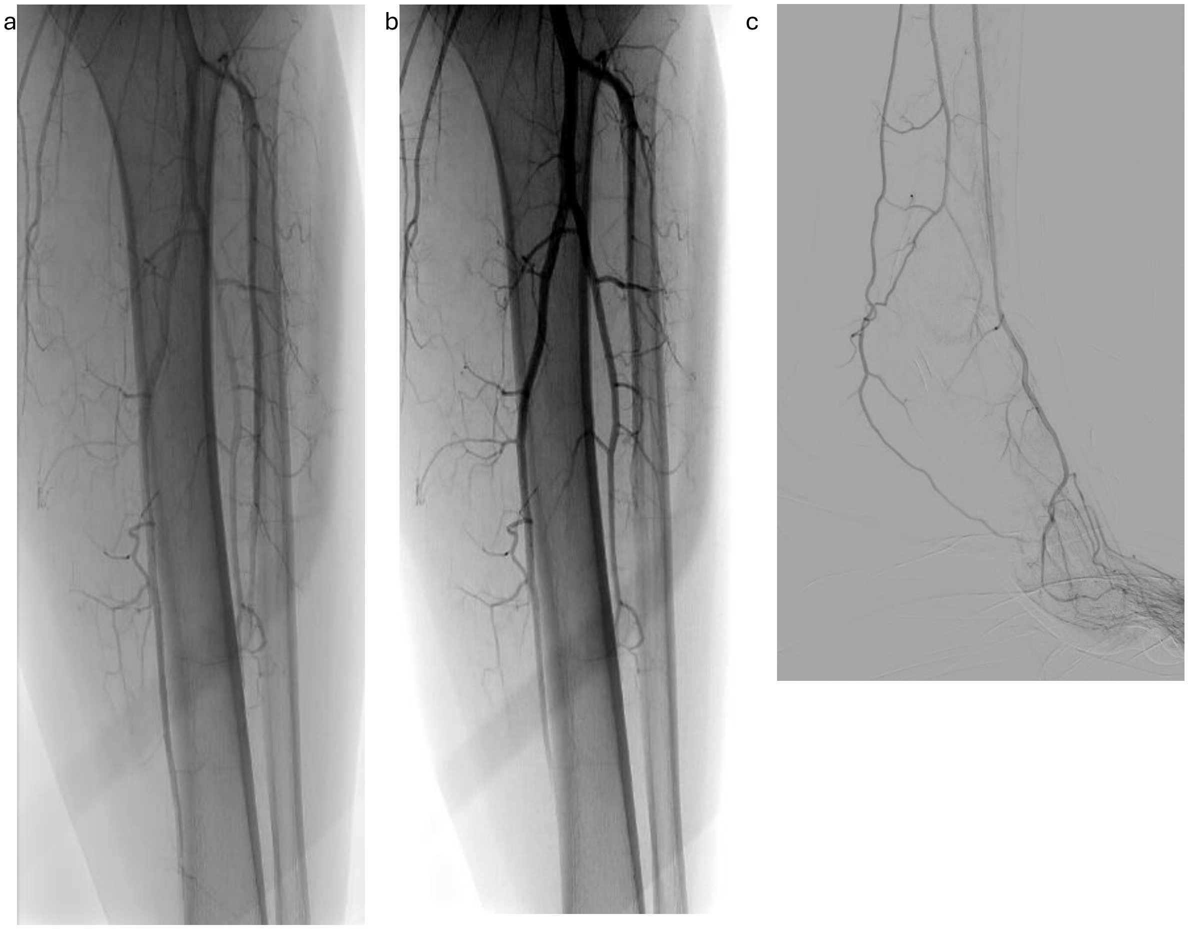

Figure S2. Angiographic evaluation pre and post MABs infusion.

a) Angiography of left lower leg before MABs infusion, showing patency of popliteal and tibial arteries; b) Angiography of left lower leg after MABs infusion, confirming patency of popliteal and tibial arteries; c) Angiography of left foot after injection confirms patency of dorsalis pedis and plantar arteries.

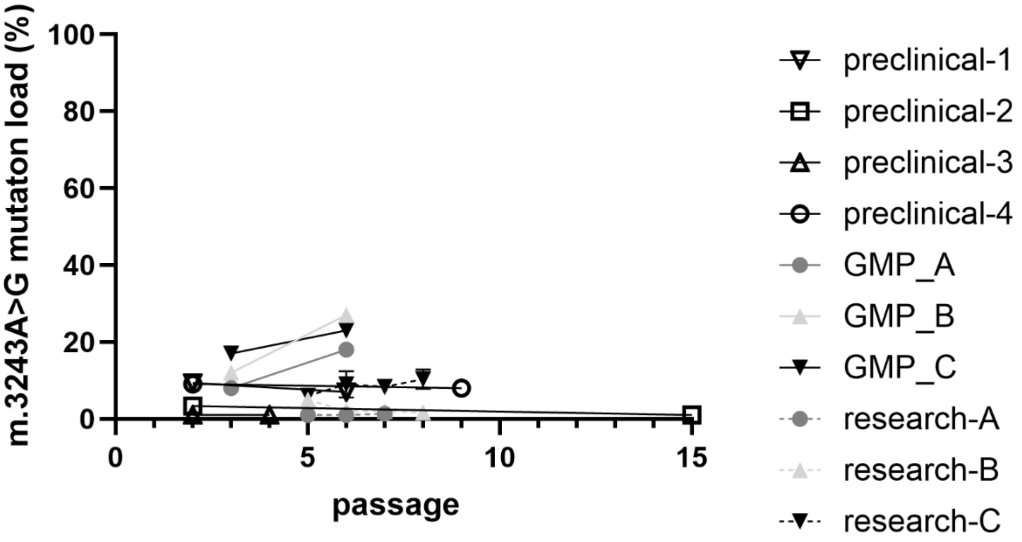

Figure S3. m.3243A>G load analysis at different passages during MABs culture.

Quantification of m.3243A>G mutation load of mesoangioblasts of participant A, B and C collected at visit 1 that were cultured in the research lab (research-), mesoangioblasts of participant A, B and C collected at visit 2 that were cultured in the GMP lab and transplanted (GMP_), and four additional mesoangioblast cultures that were analyzed in the research lab during preclinical phase (preclinical-).
